# Cancer du sein bilatéral synchrone au Maroc: caractéristiques épidémiologiques et cliniques

**DOI:** 10.11604/pamj.2015.20.118.6136

**Published:** 2015-02-10

**Authors:** Houssine Boufettal, Naïma Samouh

**Affiliations:** 1 Service de Gynécologie - Obstétrique «C», Centre Hospitalier Universitaire Ibn Rochd, Faculté de Médecine et de Pharmacie, Université Hassan 2, Casablanca, Maroc

**Keywords:** Cancer du sein bilatéral synchrone, Facteurs de risque, histologie, traitement, Pronostic, Synchronous bilateral breast cancer, risk factors, histology, treatment, prognosis

## Abstract

Préciser la fréquence, les facteurs de risque et le pronostic du cancer du sein bilatéral, à partir d'une étude rétrospective de 22 cas de cancer du sein bilatéral synchrone dans un pays du Maghreb. De 2002 à 2010, 625 patientes étaient prises en charge pour cancer du sein au service de Gynécologie-Obstétrique «C» du centre hospitalier universitaire de Casablanca. 22 cas de cancer bilatéral synchrone étaient diagnostiqués. Nos résultats sont comparés avec ceux de la littérature. La fréquence de la bilatéralité du cancer du sein synchrone était de 3,52% (22/625). L'intervalle de temps moyen entre les deux cancers est de 4 mois (0 à 6 mois). Les patientes âgées de moins de 40 ans lors du premier cancer avaient six fois plus de risque de développer un cancer au niveau du sein controlatéral que les femmes âgées de plus de 40 ans. Les patientes atteintes d'une tumeur T3 ou T4 avaient un risque neuf fois plus élevé que les autres. 90,9% (2/22) des cas des premiers cancers sont des adénocarcinomes infiltrants. Les types histologiques du premier et du douzième cancer étaient identiques dans 86,4% (19/22) des cas. Quant au pronostic, il dépend à la fois du stade du premier et du deuxième cancer et le traitement de ce dernier doit obéir aux mêmes règles du traitement du premier cancer. L'incidence du cancer bilatéral synchrone du sein est de 3,52% dans notre série. Le cancer du sein unilatéral constitue un facteur de risque de développement d'un cancer du sein controlatéral. Une surveillance à vie est nécessaire au cours d'un cancer du sein pour détecter un cancer controlatéral.

## Introduction

Le cancer du sein bilatéral synchrone était une pathologie assez fréquente. Son incidence varie selon les auteurs de 1.5 à 3.5% et selon l'intervalle diagnostique choisi par les auteurs [1–13]. Pour la majorité d'entre eux, la deuxième tumeur devait être diagnostiquée dans les six mois. Les femmes traitaient pour un cancer du sein unilatéral sont à haut risque (cinq à sept fois) de développer un nouveau cancer au niveau du sein controlatéral [1–7]. Ce facteur de risque, justifiait la surveillance à vie de toute femme traitait pour un cancer du sein, par un examen clinique et une mammographie annuelle [5]. La prise en charge thérapeutique des cancers du sein bilatéral synchrone discutait et variable selon les équipes et les périodes. Le geste chirurgical local proposait aux patientes était souvent radical (mastectomie), au détriment du traitement conservateur [9]. De plus, les techniques d’évaluation de l'envahissement ganglionnaire axillaire étaient en pleine évolution et l'intérêt de la technique du ganglion sentinelle dans cette indication était un atout majeur [10]. La physiopathologie de cette entité discutait. S'agit-il de deux événements successifs d'une même tumeur primitive d'origine monoclonale ou bien deuxième tumeur primitive indépendante? Notre objectif était de préciser la fréquence et les facteurs de risque de la récidive controlatérale du cancer du sein dans une population de femmes marocaines, prises en charge pour cancer du sein bilatéral et synchrone, à travers une série de 22 cas et une revue de la littérature.

## Méthodes

Il s'agit d'une étude rétrospective concernant les cas de cancer du sein bilatéral synchrone pris en charge au service de Gynécologie - Obstétrique «C» du centre hospitalier universitaire Ibn Rochd de Casablanca durant neuf ans, de 2002 à 2010. Durant cette période, 625 patientes étaient traitées pour cancer du sein. Parmi elles, 22 avaient un cancer du sein bilatéral synchrone. Les patientes ayant développées des métastases étaient exclues de l’étude. Nous procédons à l'analyse des données épidémiologiques, cliniques, mammographique, histologiques et pronostiques. Le dépistage du cancer du sein controlatéral était basé sur trois éléments: l'autopalpation du sein controlatéral, l'examen clinique tous les trois mois les deux premières années puis tous les six mois et une mammographie de contrôle annuelle; la scintigraphie osseuse réalisait à la demande. Un deuxième cancer du sein opposé a été défini par un cancer histologiquement prouvé et la certitude de l'absence de localisation métastatique en dehors du sein durant l'intervalle séparant les deux cancers et au moment du diagnostic du dernier cancer. Le cancer bilatéral du sein considérait comme synchrone si le deuxième cancer a été découvert au même temps ou durant un intervalle inférieur à six mois. Pour chaque patiente, la tumeur était classée selon la classification TNM de l′UICC (Union for International Cancer Control) et de l′AJC (American Joint Committee on Cancer) de 2002. Le test Chi2 a utilisait pour comparer les résultats.

## Résultats

Notre étude concernait 22 patientes traitées pour un cancer du sein bilatéral synchrone durant la période d’étude de neuf ans. Durant la même période d’étude, 625 patientes étaient traitées pour cancer du sein. L'incidence du cancer du sein bilatéral synchrone dans notre service est ainsi de 3.5%. L’âge de nos patientes variait entre 30 et 68 ans avec une moyenne d’âge de 43 ans. Les femmes âgées de moins de 40 ans avaient six fois plus de risque de développer un cancer controlatéral que celles dont l’âge est supérieur à 40 ans (p < 0,05) (Tableau 1). Les nullipares représentaient 36.3% des cas (8/22), les paucipares 13.6% des cas (3/22) et les multipares 50% des cas (11/22). Douze patientes étaient en période d'activité génitale. Sept étaient ménopausées, soit 36.8%. L’âge de la ménopause était en moyenne de 48 ans, avec des extrêmes de 42 à 51 ans. Aucune patiente n'avait reçu un traitement hormonal substitutif de la ménopause dans cette série. L’âge des patientes au moment du diagnostic du deuxième cancer au niveau du sein opposé variait de 32 à 59 ans avec un âge moyen de 43 ans. Le diagnostic du cancer du sein opposé était fait dans des circonstances variées. Dans six cas, il était découvert grâce à l'autopalpation du sein pendant l'intervalle entre les visites. Dans 16 cas, cette découverte était faite par l'examen effectué lors de la visite de contrôle ainsi que par la mammographie. Aucune de nos patientes n'avait bénéficié d'imagerie par résonance magnétique. La taille de la tumeur du premier cancer variait de un à sept centimètres avec une moyenne de 3,8 cm. La tumeur était classée T1 dans 13.6% (3/22), T2 dans 22.7% (5/22), T3 dans 40.9% (9/22) et T4 dans 22.7% (5/22) (Tableau 2).

**Tableau 1:** Montrant la répartition du cancer bilatéral du sein dans notre série selon l’âge

Tranche d’âge (ans)	Nombre	Pourcentage (%)
30 - 39	3	13.6
40 - 49	10	45.4
50 - 59	5	22.7
60 - 69	4	18.2
**Total**	22	100

**Tableau 2:** Montrant la répartition des cancers du sein bilatéraux synchrones de notre série selon l'envahissement local

	Premier cancer		Deuxième cancer	
	Nombre	Pourcentage	Nombre	Pourcentage
T1	3	13.6	5	22.7
T2	5	22.7	11	50
T3	9	40.9	4	18.2
T4	5	22.7	2	9.1
Total	22	100	22	100

Le quadrant supéro-externe était le siège de prédilection des cancers bilatéraux retrouvé dans 65% des cas au premier cancer et 48% au douzième cancer. Les récidives controlatérales prédominaient chez les femmes dont la tumeur initiale était située au niveau du quadrant supéro-externe: 78% des cas contre 23% pour les autres localisations. Cinq patientes avaient un cancer en poussée évolutive au premier cancer, ce qui représentait 22.7% des cas. Aucun cas de cancer inflammatoire n’était observé parmi les cancers du sein controlatéral. Le carcinome canalaire infiltrant (CCI) était le type histologique le plus fréquemment observé et ce dans 20 cas (90.9%). Le carcinome lobulaire était retrouvé dans trois cas (13.6%). Vingt une patientes avaient le même type histologique pour les deux seins soit 95.4%; 19 avaient un carcinome canalaire infiltrant bilatéral soit 86.3% et deux patientes avaient un carcinome lobulaire infiltrant bilatéral soit 9.1% (Tableau 3). Le type histologique était différent par rapport aux deux seins chez une patiente soit 4.5% qui avait un carcinome lobulaire infiltrant au niveau du sein gauche et un carcinome canalaire infiltrant au niveau du sein droit. L'envahissement ganglionnaire était noté dans 77.2% des cas (17/22). L’étude du grade de Scraff Bloom et Richardson (SBR) montre qu'il s'agissait d'un grade I dans 13.6% des cas (3/22), d'un grade II dans 22.7% des cas (5/22) et d'un grade III dans 63.6% des cas (14/22). Les récepteurs aux œstrogènes étaient positifs dans 50%, ceux à la progestérone étaient notés chez 63,6% des cas. L'hercept test étudiait chez sept, Quatre patientes (4/7) avaient l'Her2neu positif soit 57.1%. Chez trois patientes parmi sept (42.8%), ce marqueur était négatif. Aucun autre marqueur (Ki27, P53, etc...) n’était étudié (Tableau 4).

**Tableau 3:** Répartition des différents types histologiques des cancers bilatéraux synchrones de notre série

	Nombre de cas	Pourcentage (%)
Carcinome canalaire infiltrant bilatéral	19	86.3
Carcinome lobulaire infilrant bilatéral	2	9.1
CCI-CLI	1	4.5
Total	22	100

(CCI: Carcinome canalaire infiltrant; CLI: Carcinome lobulaire infilrant)

**Tableau 4:** Montrant le traitement chirurgical mammaire et axillaire réalisé dans le cancer du sein bilatéral de notre série

Type de traitement chirurgical	N	%
Mastectomie bilatérale	15	68.2
Tumorectomie bilatérale	1	4.6
Mastectomie-tumorectomie	5	22.7
Curage ganglionnaire bilatéral	21	95.4
Abstention bilatérale	1	4.6
Abstention	1	4.6
Total	21	95.4

Le cancer était en poussée évolutive dans 26.3% des cas (5/22). Vingt patientes traitaient par l'association chirurgie, chimiothérapie et radiothérapie soit 90.9%. Vingt-un patientes avaient subi un geste chirurgical bilatéral, ce geste consistait en une mastectomie bilatérale chez 15 patientes, en une mastectomie unilatérale et en une tumorectomie du sein controlatéral chez cinq cas et en une tumorectomie bilatérale dans un cas. Une seule patiente n'avait subi aucun geste chirurgical, ni mammaire ni axillaire, en dehors de la biopsie chirurgicale, elle présentait une métastases à distance au moment du diagnostic. Elle était perdue de vue par la suite. Le curage axillaire bilatéral était pratiqué chez 21 patientes. Le ganglion sentinelle n’était pratiqué chez aucune patiente. Dans notre série aucune patiente n'avait bénéficié d'un geste de reconstruction mammaire (Tableau 5). La radiothérapie postopératoire était pratiquée dans 21 cas. Treize patientes avaient reçu une chimiothérapie selon le protocole AC 60. Le protocole FAC était utilisé dans neuf cas. Une chimiothérapie néoadjuvante était administré à cinq patientes (26.3% des cas) pour des tumeurs en poussée évolutive, au stade T4 ou de taille supérieure à six centimètres. L'hormonothérapie était administrée chez 11 patientes. La castration par radiothérapie était réalisée chez trois patientes. L'hormonothérapie additive était reçues chez neuf patientes, elle faisait appel aux anti-œstrogènes (Tamoxifène). Sur le plan pronostique, les métastases étaient observées chez quatre patientes, dans un délai de trois à 32 mois après le traitement du douzième cancer. Parmi ces femmes, deux étaient décédées deux ans après le diagnostic du douzième cancer. Quinze patientes étaient suivies avec un recul de sept à 46 mois après le traitement du douzième cancer. Neuf, parmi elles, n'avaient pas présenté de rechute. Six étaient perdues de vue.

**Tableau 5:** Présence ou absence des récepteurs aux œstrogènes au niveau des cancers bilatéraux du sein

	Nombre	Pourcentage (%)
Récepteurs aux E2 Positifs	11	50
Récepteurs aux E2 Négatifs	11	31.8
Récepteurs à la progestérone Positifs	14	63.6
Récepteurs à la progestérone Négatifs	8	36.4
Her 2 neu Positif	4	18.2
Her 2 neu Négatif	3	13.6
Her 2 neu Non fait	15	68.2

(E2: Estrogènes)

## Discussion

On considère que le cancer du sein est bilatéral lorsque les deux seins sont siège d′une lésion maligne [1–4]. Cette bilatéralité peut être diagnostiquée au même temps ou durant un intervalle de six mois, c'est le cancer du sein bilatéral synchrone. La bilatéralité ainsi que la multicentricité d′un cancer du sein reflète en fait la capacité de la transformation néoplasique d′apparaître à des endroits différents du tissu mammaire de façon indépendante, simultanée ou non [5–8]. Plusieurs critères permettent de distinguer un réel deuxième cancer primitif du sein d'une localisation métastatique à ce niveau [3, 9–11]. En effet, les deux cancers sont de types histologiques différents; la présence d'une composante in situ dans le cancer du sein controlatéral; le degré de différenciation du douzième cancer est plus élevé par rapport au premier cancer et l'absence de métastase lors du traitement du premier cancer avec un intervalle de temps entre les deux cancers de plusieurs années [12–17]. Cependant, il n'existe pas un consensus quant à la définition d′un cancer du sein bilatéral simultané, ce qui rend l’étude du cancer du sein bilatéral parfois difficile. Pour certains auteurs, le cancer du sein controlatéral est synchrone s′il est découvert pendant le traitement du premier cancer ou dans le mois qui suit [3, 18, 19]; pour d'autres, lorsqu'il est découvert dans les six premiers mois [14] ou les 12 mois après le traitement du premier cancer [5, 18, 20]. Les différentes séries publiées ont montré une incidence de cancer du sein bilatéral synchrone comprise entre 1.2 et 3.5% [3, 17, 21]. Dans notre série, la fréquence du cancer du sein bilatéral synchrone est de 3,52%. Plusieurs auteurs ont étudié les caractéristiques épidémiologiques des patientes présentant un cancer synchrone bilatéral du sein. Le but de ces études était de définir une population à risque susceptible de bénéficier d'une surveillance ciblée, voire d'un traitement prophylactique [22–24]. Leurs résultats sont contradictoires [25].

Certains facteurs de risque d′atteinte du sein controlatéral sont reconnus dans la majorité des publications. Ce sont: le jeune âge de la femme au moment du traitement du premier cancer (35 à 40 ans), les antécédents familiaux de cancer du sein, un premier cancer du sein de type lobulaire (in situ ou infiltrant), canalaire in situ, multifocal ou multicentrique. La corrélation avec d′autres facteurs est moins bien établie, tels que le volume et le siège du premier cancer, l′envahissement ganglionnaire et le grade histo-pronostique [6, 9, 22, 23]. Cependant, le cancer génétique n'est pas considéré comme un facteur de risque d'atteinte du sein controlatéral [21]. L’âge jeune de patientes ainsi que le statut pré ménopausique sont des facteurs de risque parfois cités dans la littérature [24, 25]. Dans notre étude, l’âge moyen des patientes ayant un cancer synchrone bilatéral du sein était de 43 ans. Cependant, il existait une évolution dans le temps avec un rajeunissement de notre population. Dans la majorité′ des études récentes et notamment celles comparant les cancers synchrones bilatéraux du sein aux cancers du sein unilatéraux [20, 21, 24], le jeune âge et le statut pré ménopausique ne sont pas considérés comme des facteurs de risque de cancer du sein bilatéral. Le risque d′apparition d′un cancer du sein controlatéral est marqué quand l′âge de la femme est inférieur à 40 ans au moment du traitement du premier cancer. Pour ce groupe de femmes, le risque est de cinq à 10 pour 1000 femmes par années [20, 23]. Par contre, ce risque est plus faible si le premier cancer est traité à un âge post-ménopausique où il est inférieur à quatre pour 1000 femmes par années [22]. Pour Thomas [17], les femmes ayant un cancer du sein unilatéral à un âge inférieur à 40 ans ont trois fois plus de risque de développer un second cancer controlatéral que celles ayant un cancer unilatéral à un âge supérieur à 40 ans [7, 15]. Dans notre étude, les femmes de moins de 40 ans avaient six fois plus de risque d'avoir un deuxième cancer au niveau du sein opposé que les femmes plus âgées. Les antécédents familiaux de cancer du sein sont fréquents chez les patientes présentant un cancer du sein bilatéral synchrone [24]. La présence d′antécédents familiaux de cancer du sein est un facteur de risque important d′apparition d′un cancer du sein controlatéral. La probabilité d′avoir un cancer du sein controlatéral est, dans certaines études, de 28% parmi les femmes ayant des antécédents familiaux de cancer du sein, contrastant avec une probabilité de 13% pour les femmes sans antécédents familiaux [19]. Le risque de bilatéralité augmente davantage si, au facteur familial, s′associe le jeune âge de la patiente au moment du diagnostic du premier cancer [4–20].

La probabilité atteint 35% à la dix-neuvième année chez les patientes avec antécédents familiaux de cancer du sein et qui étaient âgées de moins de 50 ans lors de leur premier cancer. Dans notre série, la notion d'antécédents familiaux n'a été observée dans aucun cas de cancer du sein bilatéral synchrone. Concernant le type histologique du premier cancer du sein, la plupart des études montrent qu′un cancer lobulaire in situ ou infiltrant est lié à un risque accru de survenue d′un cancer du sein controlatéral. Le carcinome canalaire in situ joue un rôle modeste dans le risque de bilatéralité [25]. Dans notre série, le carcinome canalaire infiltrant a été retrouvé dans 90.9% des cas; alors que le type lobulaire infiltrant a été retrouvé dans 9.1% cas. Le douzième cancer du sein était du même type histologique que le premier cancer dans tous les cas que nous avons observé. Le caractère multifocal ou multicentrique du premier cancer primitif du sein est fortement lié au risque de bilatéralité [13, 23]. Irvine [21] retrouve une forte proportion de bilatéralité chez les patientes ayant un cancer multicentrique et de type lobulaire in situ. D′autres facteurs tels que le stade, le volume tumoral, le siège, l′envahissement ganglionnaire et le grade histopronostique pourraient avoir un rôle dans l′apparition d′un cancer du sein controlatéral [20]. Dans notre étude, le risque de cancer bilatéral synchrone augmente avec l′importance du volume tumoral du premier cancer. En revanche, nous n'avons pas trouvé de corrélation en ce qui concerne le statut ganglionnaire puisque presque tous les premiers cancers s'accompagnaient d'un envahissement ganglionnaire. Ben Hassouna [5] ne trouve pas de corrélation significative entre la bilatéralité et synchronicité et le siège du premier cancer, le grade histopronostique ou l′état ganglionnaire. Dans notre série, le siège du premier cancer primitif était dans 65% des cas le quadrant supéro-externe. D'autres études n'ont pas objectivé de différence significative par rapport au premier cancer concernant les récepteurs hormonaux, l'expression de l'HER2 ou du p53 [24, 25]. L′irradiation des seins est un autre facteur de risque d′atteinte bilatérale. Selon la littérature, le risque de survenue d′un cancer mammaire après traitement de la maladie de Hodgkin serait de 6 à 9% [20, 21]. Les facteurs de risque sont l′irradiation et le jeune âge au moment du traitement initial. Par conséquent, les filles et les jeunes femmes traitées pour maladie de Hodgkin, doivent être surveillées rigoureusement par des examens cliniques et mammographiques répétés [20].

La plupart des études ont montré qu'un deuxième cancer primitif du sein opposé bien traité n'altère pas le pronostic de façon significative. La présence de facteurs de haut risque de bilatéralité (âge très jeune, antécédents familiaux de cancer du sein ou cancer multifocal) ne justifie pas de geste sur le sein controlatéral en l'absence de lésion suspecte à l'examen clinique ou à la mammographie [21]. La mastectomie prophylactique du sein controlatéral utilisée par certaines équipes n'a pas permis d'améliorer le pronostic [22]. Le suivi correct des patientes ayant un cancer du sein unilatéral, permet de découvrir le deuxième cancer à un stade plus précoce, offrant la possibilité d'un traitement aussi efficace que dans le premier cancer [23]. D'autant plus que la survenue d'un deuxième cancer du sein controlatéral n'est pas une contre-indication au traitement conservateur. La comparaison avec les patientes traitées de la même façon pour un cancer du sein unilatéral de même stade a retrouvé un taux de survie à cinq ans qui est le même et un résultat esthétique identique dans les deux groupes. Tout cela permet d'annoncer que le traitement du cancer du sein bilatéral doit être envisagé vis-à-vis de chaque sein à part. Concernant les données sur le pronostic et l'impact de la survenue du 2^e^ cancer au niveau du sein controlatéral, les données sont divergentes [25]. Pour certains auteurs, le taux de survie après cancer du sein bilatéral est le même que celui des patientes traitées pour un cancer du sein unilatéral, à stade égal. D'autres rapportent un pronostic moins bon. Maarse et al. [20] rapportent une survie à cinq ans sans récidive et sans métastase de 94% dans le groupe du cancer du sein unilatéral et de 91% dans celui du cancer du sein bilatéral, sans aucune différence significative (p = 0,16). Cependant, le pronostic des patientes traitées pour cancer du sein bilatéral dépend des éléments pronostiques à la fois du premier et du deuxième cancer [24].

## Conclusion

Les cancers synchrones bilatéraux du sein ne sont pas exceptionnels, ils représentent 3.52% des cancers du sein. Si l'antécédent personnel de cancer du sein est le principal facteur de risque de survenue d'un cancer controlatéral, la présence d'une tumeur multifocale ou d'un carcinome lobulaire infiltrant, doivent mener à une exploration précise du sein controlatéral. L'IRM, examen qui a permis de diagnostiquer un cancer controlatéral infra clinique sans traduction en imagerie conventionnelle. Chaque tumeur doit donc être traitée comme un cancer unique, en proposant, lorsqu'il est indiqué, un traitement conservateur. La technique du ganglion sentinelle, est un atout majeur dans la prise en charge thérapeutique des cancers synchrones bilatéraux du sein. Les modalités thérapeutiques du cancer du sein opposé sont les mêmes que celles du premier cancer du sein. Une surveillance à vie des patientes traitées pour un cancer du sein unilatéral permet de détecter plus précocement un cancer du sein controlatéral lui offrant ainsi des possibilités thérapeutiques et un pronostic meilleurs.
